# Steroid Sulfatase Regulates Metabolic Reprogramming in Advanced Prostate Cancer

**DOI:** 10.3390/cancers17121959

**Published:** 2025-06-12

**Authors:** Masuda Sharifi, Cameron M. Armstrong, Shu Ning, Amy R. Leslie, Zachary A. Schaaf, James P. Maine, Wei Lou, Pui-Kai Li, Hongyu Xu, Chengfei Liu, Allen C. Gao

**Affiliations:** 1 Department of Urologic Surgery, University of California at Davis, Davis, CA 95616, USA; 2 Division of Medicinal Chemistry and Pharmacognosy, College of Pharmacy, The Ohio State University, Columbus, OH 43210, USA; 3 Department of Oncology, 363 Hospital, Chengdu 610041, China; 4 UC Davis Comprehensive Cancer Center, University of California at Davis, Sacramento, CA 95616, USA; 5 VA Northern California Health Care System, Sacramento, CA 95655, USA

**Keywords:** prostate cancer, steroid sulfatase, therapeutic resistance, metabolic reprogramming

## Abstract

Background/Objective: The expression of human steroid sulfatase (STS) is upregulated in castration-resistant prostate cancer (CRPC) and is associated with resistance to anti-androgen drugs, such as enzalutamide (Enza) and abiraterone (Abi). Despite the known link between STS overexpression and therapeutic unresponsiveness, the mechanism by which STS confers this phenotype remains incompletely understood. In this study, we sought to understand how STS induces treatment resistance in advanced prostate cancer (PCa) cells by exploring its role in altering mitochondrial activity. Methods: To examine the effects of increased STS expression on mitochondrial respiration and programming, we performed RNA sequencing (RNA-seq) analysis, the Seahorse XF Mito Stress Test, and a mitochondrial Complex I enzyme activity assay in STS-overexpressing cells (C4-2B STS) and in enzalutamide-resistant CPRC cells (C4-2B MDVR). We employed SI-2, the specific chemical inhibitor of STS, on C4-2B STS and C4-2B MDVR cells and evaluated STS activity inhibition on mitochondrial molecular pathways and mitochondrial respiration. Lastly, we examined the effects of dehydroepiandrosterone sulfate (DHEAS) supplementation on C4-2B STS organoids. Results: We present evidence from the transcriptomic profiling of C4-2B STS cells that there are enriched metabolic pathway signatures involved in oxidative phosphorylation, the electron transport chain, and mitochondrial organization. Moreover, upon STS inhibition, signaling in the electron transport chain and mitochondrial organization pathways is markedly attenuated. Findings from the Seahorse XF Mito Stress Test and mitochondrial Complex I enzyme activity assay demonstrate that STS overexpression increases mitochondrial respiration, whereas the inhibition of STS by SI-2 significantly reduces the oxygen consumption rate (OCR) and Complex I enzyme activity in C4-2B STS cells. Similarly, an increased OCR and electron transport chain Complex I enzymatic activity are observed in C4-2B MDVR cells and a decreased OCR upon SI-2 inhibition. Lastly, we show that STS overexpression promotes organoid growth upon DHEAS treatment. Conclusions: Our study demonstrates STS as a key driver of metabolic reprogramming and flexibility in advanced prostate cancer. Disrupting enhanced mitochondrial respiration via STS presents a promising strategy in improving CRPC treatment.

## Simple Summary

Many prostate cancer patients with late-stage disease are non-responsive to current treatments. One potential mechanism is that advanced cancer cells can alter their metabolism to overcome therapeutic attack through steroid sulfatase activity, a key enzyme of the steroid hormone metabolic pathway. This study investigates how steroid sulfatase modifies mitochondrial programming in treatment-resistant prostate cancer cells. Here, we observe increased mitochondrial activity and an increased cellular oxygen consumption rate in steroid sulfatase overexpressing cells, whereas steroid sulfatase inhibition reduced mitochondrial functionality. Our study demonstrates that disrupting enhanced mitochondrial respiration, driven by steroid sulfatase, could provide a strategy for improving drug resistance in advanced prostate cancer.

## 1. Introduction

Prostate cancer (PCa) is one of the major causes of cancer-related deaths amongst American men, with androgen deprivation therapy (ADT) as a main, initial treatment [1,2,3]. Although this approach is effective at first, most patients gain only temporary benefits before developing castration-resistant prostate cancer (CRPC), a more aggressive form of the disease with limited treatment options [4,5]. Next-generation anti-androgen therapies (NGATs), such as enzalutamide (Enza), abiraterone (Abi), apalutamide (Apal), and darolutamide (Daro), have proved effective at treating CRPC by targeting the androgen receptor (AR) or androgen synthesis, but resistance to these therapies is still a significant challenge [6,7]. Treatment failure and disease progression are often linked to the AR and the androgen signaling axis. Evasive mechanisms include increased AR expression and/or amplification, alternative AR ligand-binding activity, AR splice variants, and intra-tumoral androgen production involving alternative pathways to androgen synthesis [4,8].

One candidate of continued androgen signal transduction and NGAT resistance is steroid sulfatase (STS), an enzyme that hydrolyzes steroid sulfates to mediate the production of dehydroepiandrosterone (DHEA) from its conjugated form, dehydroepiandrosterone sulfate (DHEAS) [9]. We previously showed that upon supplementation with DHEAS, CRPC cells upregulating STS expression and activity have increased intracellular androgen levels, such as DHEA [10]. Given that DHEAS and its active form, DHEA, are the most abundantly present steroids in human circulation, they have been documented to serve diverse physiological roles, including in oxidative energy metabolism [11,12,13]. For example, in the cumulus cells of aging women, DHEA administration rescues decreased mitochondrial oxygen consumption and turnover rates by increasing cellular energetic output [14]. In human cervical cancer, STS overexpression in HeLa cells is linked to enhanced aerobic glycolysis via the upregulation of hypoxia-inducible factor 1-alpha expression [15]. In the rat brain, DHEA’s roles involve modulating glucose uptake and breakdown to increase mitochondrial respiration, resulting in enhanced metabolic energy [12,16]. Taken together, STS and its downstream metabolite, DHEA, are critical in altering energy metabolism.

It is well recognized that during PCa advancement, cells undergo metabolic reprogramming to switch towards enhanced oxidative phosphorylation for cellular survival and growth [17,18,19,20]. However, the effect of STS activity on metabolic flexibility and cellular respiration in NGAT resistance and CRPC development has not yet been determined. In this study, we found that STS overexpression and enzalutamide-resistant CRPC cells exhibit enhanced mitochondrial respiration and Electron Transport Chain Complex I activity in comparison to control cells. Upon STS inhibition with a specific chemical inhibitor, SI-2, we found significantly suppressed oxidative phosphorylation capacity in the same STS overexpression and enzalutamide-resistant CRPC cell lines. Furthermore, we demonstrated that DHEAS supplementation enhances STS overexpression and PDX-derived organoid growth. Altogether, we showed that targeting STS could be a potential strategy to combat CRPC progression and therapeutic resistance by disrupting STS-regulated metabolic pathways and programming.

## 2. Materials and Methods

### 2.1. Cell Culture

C4-2B Neo and C4-2B STS prostate cancer cells were previously generated by the stable transfection of C4-2B cells with STS-encoding plasmids from GenScript (Piscataway, NJ, USA), alongside an empty vector [10]. Lipofectamine was used as a transfection reagent, and Western blot analysis confirmed STS overexpression as previously described. C4-2B Neo and C4-2B STS cells were maintained in RPMI 1640 medium containing 10% fetal bovine serum (FBS), 100 units per ml of penicillin, 0.1 mg per ml of streptomycin, and 300 μg/mL of G418. Cell lines were routinely tested for mycoplasma by aMycoplasma PCR Detection Kit (Cat #: G238) purchased from Applied Biological Materials (Richmond, BC, Canada). Cultured cells were maintained in a 37 °C humidified incubator with 5% carbon dioxide. Enzalutamide-resistant C4-2B cells (MDVR) were generated as previously described and were maintained in RPMI 1640 medium containing 10% fetal bovine serum (FBS), 100 units per ml of penicillin, 0.1 mg per ml of streptomycin, and 20 µM of enzalutamide [21]. Enzalutamide (Cat#: S1250) was purchased from Selleck-chem (Houston, TX, USA).

### 2.2. Seahorse Extracellular Flux Analyzer Experiments

Agilent Seahorse XF Cell Mito Stress Tests (Seahorse, Agilent Technologies, Santa Clara, CA, USA) were performed on the Seahorse XFe24 extracellular flux analyzer in accordance with the manufacturer’s instructions [22,23]. Cells subjected to SI-2 were treated with various concentrations of SI-2 for 2 days prior to running the assay. The day prior to the assay, adherent cells were seeded in a Seahorse culture plate at a density of 26,000 cells/well in RPMI 1640 medium containing 10% fetal bovine serum (FBS), 100 units per mL of penicillin, and 0.1 mg per mL of streptomycin. The Seahorse sensor cartridge was hydrated with 1 mL of XF calibrant the day prior to assay and placed in a non-CO_2_ 37 °C incubator overnight. On the day of the assay, XF assay medium was warmed to 37 °C in a water bath and supplemented with 10 mM glucose, 1 mM pyruvate, and 2 mM glutamine. Compounds modulating respiration were added into the cell wells during assay at the following final concentrations: (1) 15 μM of oligomycin; (2) 20 μM of carbonyl cyanide-4-(trifluoromethoxy) phenylhydrazone (FCCP); (3) 5 μM of rotenone and Antimycin. Data was normalized by protein concentration and analyzed using the Seahorse XF Wave software (Version 2.6) and the Seahorse XF Mito Stress Test Report Generator.

### 2.3. Electron Transport Chain Complex I Activity

The analysis of mitochondrial Complex I enzyme activity was performed using the Complex I Enzyme Activity Assay Kit (Colorimetric)(Cat #: ab109721) from Abcam (Cambridge, UK). Two days prior to the assay, adherent cells were plated in 10 cm Petri dishes at a density of 2,000,000 cells/plate and two plates per group. Cells subjected to SI-2 were treated with various concentrations of SI-2 for 2 days prior to running the assay. On the day of assay, adherent cell pellets were resuspended in PBS, and protein concentrations were determined. Samples were then treated with detergent, and protein concentrations were determined again. Cell extracts were diluted to a final concentration of 250 µg/mL. The optical density of samples was determined at 450 nm in kinetic mode for 30 min at room temperature. The change in absorbance at 450 nm over time, per µg of sample concentration, was calculated, and the percentage of Complex I activity is displayed as a percentage of the control sample. Data was normalized by protein concentration.

### 2.4. Organoids Culture

C4-2B Neo- and C4-2B STS cell xenograft-derived tumor tissues were collected from SCID mice (ENVIGO, Indianapolis, IN, USA). Collagenase IV (STEMCELL) was used for tumor tissue digestion. The dissociated cells were strained with a 40 μm filter to obtain a single-cell suspension. The cells were spun in the centrifuge at 3000 RPM for 5 min for pellet formation. The supernatant was then aspirated, and the pellet was resuspended in advanced ADMEM complete medium containing GlutaMAX (Gibco, Waltham, MA, USA), 100 units/mL of penicillin, 0.1 mg/mL of streptomycin, B27 (Gibco), N-Acetylcysteine (Thermo Scientific, Waltham, MA, USA), Human Recombinant EGF (Thermo Scientific), Recombinant FGF-10 (Invitrogen, Waltham, MA, USA), A-83–01 (Tocris, Bristol, UK), SB202190 (Bioscience, Bristol, UK), Nicotinamide (Thermo Scientific), PGE2 (Bioscience), Noggin (Thermo Scientific), and R-spondin (R & D Systems, Santa Clara, CA, USA). Upon determining the total cell number, C4-2B NEO and C4-2B STS cells were plated at a density of 6000 cells/well in a 96-well plate with a 1:3 Matrigel/complete ADMEM medium mix. Once the Matrigel complex was solidified, the ADMEM complete medium supplemented with the corresponding treatments was added to each well. The plate was kept in a 37 °C humidified incubator with 5% CO_2_ for 14 days for organoid formation. Organoids were visualized by immunofluorescence using a LIVE/DEAD^®^ Viability/Cytotoxicity Assay Kit (Thermo Scientific) according to the manufacturer’s protocol. ImageJ (Version 1.54) was used to quantify the total fluorescence intensity of live cells. For the C4-2B STS treated with DHEAS and various concentrations of SI-2 assay, organoids were derived from 2D-cultured C4-2B STS cells. Cells were plated in the same conditions as cell xenograft-derived organoids.

### 2.5. Cell Viability Assay

C4-2B Neo and C4-2B STS cells were plated at a density of 2000 cells/well in 96-well plates in RPMI 1640 medium containing 10% fetal bovine serum (FBS), 100 units per ml of penicillin and 0.1 mg per ml of streptomycin. The subsequent day, cells were treated with DHEAS (100 nM) and SI-2 at various concentrations (2.5 μM, 5 μM, 10 μM). Cell growth was determined by CCK-8 reagent (Dojindo, Kumamoto, Japan), according to the manufacturer’s protocol, 5 days post treatment. The cell viability rate (%) was calculated using the following formula: Cell viability rate (%) = (Treatment group cell number/Control group cell number) × 100%.

### 2.6. Colony Formation Assay

C4-2B STS cells were plated at 1200 cells/well in 6-well plates in complete RPMI 1640 medium containing 10% fetal bovine serum (FBS), 100 units per ml of penicillin and 0.1 mg per ml of streptomycin. The subsequent day, cells were treated with DHEAS (100 nM) and SI-2 at various concentrations (2.5 μM, 5 μM, 10 μM). Colonies formed for 14 days, and upon completion, colonies were fixed and stained with 0.05% *w*/*v* crystal violet, 1% of 37% formaldehyde, 1% methanol, and 1X PBS. Colony plates were then rinsed with 1X PBS the next day and allowed to air dry. Colonies were counted, and the data is displayed as a percentage of the control.

### 2.7. RNA-Seq

Total RNA extraction, using Trizol reagent (Invitrogen, Waltham, MA, USA), was performed on C4-2B NEO and C4-2B STS cells. Single-replicate RNA samples were isolated. Subsequently, extracted RNA was used for indexed RNA-Sequencing (RNA-Seq) library preparation using the KAPA Stranded mRNA-Seq Library Kit (Kapa Biosystems, Wilmington, MA, USA), in accordance with the manufacturer’s protocol. Multiplex sequencing (2 × 150 bp, paired-end, ~30 × 106 reads per sample) on the Illumina HiSeq 4000 System (Illumina, San Diego, CA, USA) was then completed by combining indexed libraries. Lastly, data analysis was accomplished with a HISAT-StringTie-Cuffnorm pipeline for the mapping/alignment of raw sequence reads (FASTQ format) to the reference human genome assembly (GRCh38/hg38). The quantification of transcript expression was conveyed as FPKM (fragments per kilobase per million fragments mapped).

### 2.8. Gene Set Enrichment Analysis

To determine transcriptomic differences between the C4-2B NEO and C4-2B STS cells, RNA-seq results were subjected to Gene Set Enrichment Analysis (GSEA). GSEA software (Version 4.1.0) from the Broad Institute was used to identify biological pathways based on the Molecular Signature Database (MSigDB). A normalized enrichment score (NES) and a nominal *p* value lower than 0.05 and an FDR q value lower than 0.25 were considered to be significant. Heatmaps were generated from the FPKM value of genes by using GSEA desktop software (Version 4.1.0). Red represents the maximum value for each gene, and blue represents the minimum value for each gene.

### 2.9. Statistical Analysis

Raw data was analyzed in GraphPad Prism 9.0. Differences between the two groups were analyzed by a two-tailed Student’s *t* test. For multiple group comparisons, one-way analysis of variance (ANOVA) was used. A *p*-value of less than 0.05 was considered significant (* *p* < 0.05, ** *p* < 0.01, *** *p* < 0.005, and **** *p* < 0.001).

## 3. Results

### 3.1. Metabolic Signaling Pathways Are Enriched in STS-Overexpressing Prostate Cancer Cells

**Figure 1:**
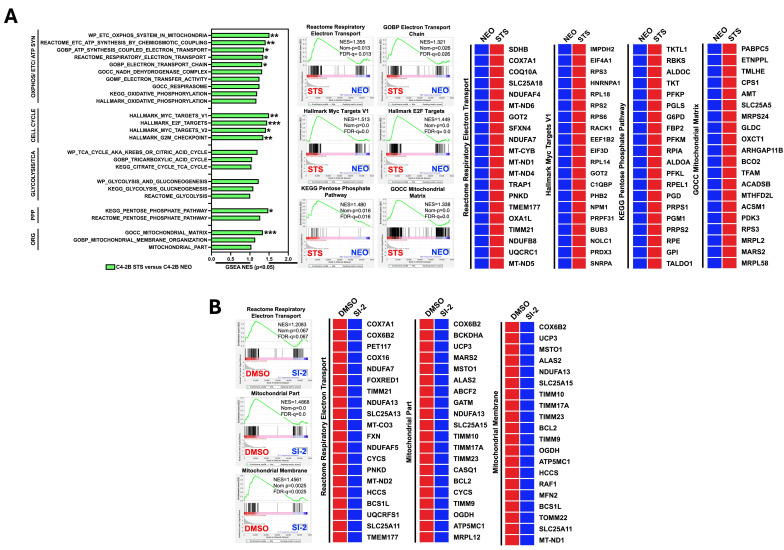
Mitochondrial metabolic pathways and cell cycle signaling are regulated by STS. (**A**) GSEA of C4-2B STS cells compared to C4-2B NEO (control) cells. Gene set collection includes pathways involved in the following: oxidative phosphorylation and the electron transport chain (OXPHOS/ETC/ATP SYN), cell cycle, glycolysis and the citric acid cycle (Glycolysis/TCA), the pentose phosphate pathway (PPP), and mitochondrial organization (ORG). GSEA enrichment plots demonstrate the significant enrichment of signaling in the Reactome Respiratory Electron Transport, GOBP Electron Transport Chain, Hallmark Myc Targets V1, Hallmark E2F Targets, KEGG Pentose Phosphate Pathway, and GOCC Mitochondrial Matrix pathways in C4-2B STS cells. The corresponding heatmaps reveal the top 20 upregulated genes in the Reactome Respiratory Electron Transport, Hallmark Myc Targets V1, KEGG Pentose Phosphate Pathway, and GOCC Mitochondrial Matrix pathways in C4-2B STS cells. Red represents the maximum value for each gene, and blue represents the minimum value for each gene. (**B**) GSEA enrichment plots of SI-2-treated (10 μM) C4-2B STS cells. GSEA plots with summary statistics for the following: Reactome Respiratory Electron Transport, Mitochondrial Part, and Mitochondrial Membrane. The heatmaps demonstrate the top 20 downregulated genes in the Reactome Respiratory Electron Transport, Mitochondrial Part, and Mitochondrial Membrane pathways upon 10 μM of SI-2 treatment in C4-2B STS cells. Red represents the maximum value for each gene, and blue represents the minimum value for each gene. (* *p* < 0.05, ** *p* < 0.01, and *** *p* < 0.005).

We previously generated STS-overexpressing cells (C4-2B STS) by the stable transfection of STS-expressing plasmids into parental C4-2B cells [10]. To dissect the molecular changes associated with STS overexpression, we performed RNA-seq analysis of C4-2B STS and control cells (C4-2B NEO). As shown in Figure 1A, mitochondrial metabolic pathways involved in the electron transport chain, oxidative phosphorylation, glycolysis, the citric acid cycle, the pentose phosphate pathway, and mitochondrial organization are significantly enriched in C4-2B STS cells compared to C4-2B NEO cells. Additionally, genes encoding several of the classic Myc target gene sets, such as HALLMARK_MYC_TARGETS_V1 and HALLMARK_MYC_TARGETS_V2, were significantly upregulated in C4-2B STS cells compared to C4-2B NEO cells (Figure 1A). Furthermore, the HALLMARK_E2F_TARGETS and HALLMARK_G2M_CHECKPOINT gene sets were significantly enriched in C4-2B STS cells, suggesting that Myc signaling activation and cell cycle progression may emerge from STS overexpression (Figure 1A). To determine the potential activity of STS, we treated C4-2B STS cells with SI-2 and performed transcriptomic analysis. We found that gene programs regulating the electron transport chain and mitochondrial structure were decreased upon STS inhibition (Figure 1B). Collectively, these results suggest that STS expression and activity impacts mitochondrial functionality and architecture.

### 3.2. STS Overexpression Enhances OXPHOS Electron Transport Chain Complex I Activity

**Figure 2:**
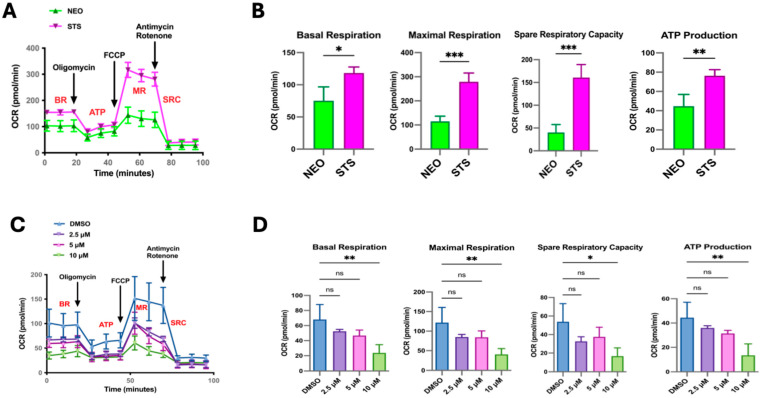
STS overexpression alters mitochondrial activity. (**A**) Using the Seahorse Extracellular Flux analyzer, the Mito Stress Test output of C4-2B NEO (NEO) and C4-2B STS (STS) cells is displayed as the OCR after the injection of respiration modulating compounds, oligomycin, carbonyl cyanide-4-(trifluoromethoxy) phenylhydrazone (FCCP), rotenone, and Antimycin. NEO is the control cell line. (**B**) Quantification of relative OCR levels in C4-2B NEO and C4-2B STS cells shown as basal respiration (BR), maximal respiration (MR), spare respiratory capacity (SRC), and ATP production (ATP). (**C**) Mito Stress Test output demonstrating mitochondrial respiration changes in C4-2B STS cells treated with SI-2 (2.5 μM, 5 μM, and 10 μM, respectively). (**D**). Relative values of BR, MR, SRC, and ATP derived from the overall OCR of C4-2B STS cells treated with SI-2 (2.5 μM, 5 μM, and 10 μM, respectively). * *p* < 0.05, ** *p* < 0.01, and *** *p* < 0.005.

Metabolic reprogramming in cancer cells is essential for proliferation and resistance to therapeutics [24,25]. This alteration in metabolism is regulated by many oncogenic factors, including the Myc family [25,26,27,28]. Given that our C4-2B STS cells have an increased expression in metabolic pathways, such as the electron transport chain and oxidative phosphorylation, we sought to determine how STS overexpression modulates mitochondrial respiration. We first assessed the oxygen consumption rates (OCRs) of the C4-2B STS (STS) and C4-2B Neo (NEO) cells by running a Mito Stress Test with the Seahorse XFe24 extracellular flux analyzer. During this assay, compounds modulating respiration are added to the cells. This allows for the measurement of basal OCR (BR), maximal respiration (MR), and spare respiratory capacity (SRC), which is the absolute difference between maximal and basal OCR. The SRC indicates a cell’s energetic durability when faced with an increased energy demand or stress conditions. In comparison to NEO cells, STS cells demonstrated higher overall OCR levels (Figure 2A). Additionally, we show that STS cells have a higher capacity to respond to changes in energetic requirements, as indicated by the BR, MR, SRC, and ATP production levels (Figure 2B). We next asked whether STS inhibition in C4-2B STS cells would affect this observed enhancement in mitochondrial respiration. As expected, the inhibition of STS by SI-2 (10 μM) significantly reduced the OCR level as shown by BR, MR, SRC, and ATP production levels (Figure 2C,D).

**Figure 3:**
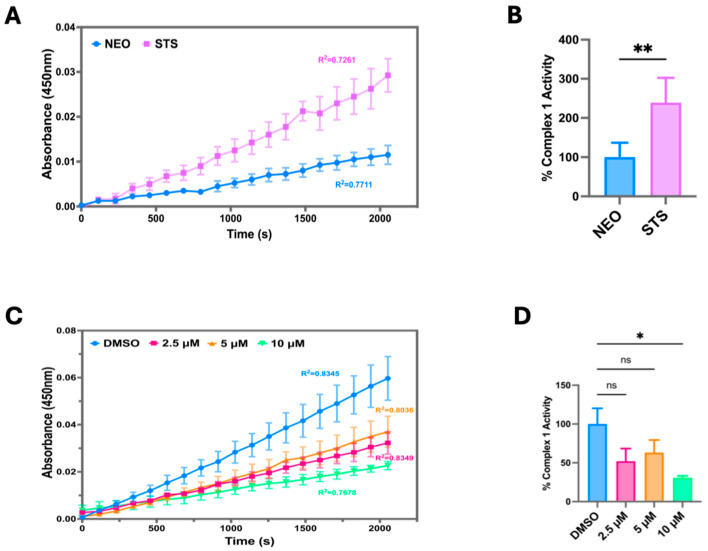
STS overexpression increases mitochondrial OXPHOS Complex I enzyme activity. (**A**) Raw Electron Transport Chain (ETC) Complex 1 enzyme activity was determined in C4-2B NEO (NEO) and C4-2B STS (STS) cell culture extracts. Samples were added to a microplate, and optical density was measured at 450 nm over time. NEO is the control cell line. (**B**) The derived % Complex 1 Activity was determined in NEO and STS cells, calculated by using the following formula: % Complex 1 Activity = (STS group linear rate of increase in absorbance at OD 450 nm over time/NEO group linear rate of increase in absorbance at OD 450 nm over time) × 100%. (**C**) Raw ETC Complex 1 activity was determined in STS cells treated with SI-2 (2.5 μM, 5 μM, and 10 μM, respectively) at OD 450 nm over time. (**D**) The slope of the linear rate of increase in absorbance at OD 450 nm over time was used to derive % Complex 1 activity in STS cells treated with SI-2 (2.5 μM, 5 μM, and 10 μM, respectively). * *p* < 0.05; ** *p* < 0.01.

To investigate how STS overexpression modulates the OCR, we measured the Electron Transport Chain (ETC) Complex I activity in C4-2B STS cells compared to control (C4-2B NEO). We found that C4-2B STS cells displayed significantly higher levels of ETC Complex I activity, suggesting that STS overexpression strongly induces oxidative phosphorylation and ETC activity (Figure 3A,B). The ETC Complex 1 contributes to the proton gradient utilized for ATP synthesis, therefore highlighting STS expression as an instrumental factor in establishing the mitochondrial proton gradient [29]. To further examine the potential role of STS in stimulating mitochondrial respiration, C4-2B STS cells were treated with SI-2 doses (2.5 μM, 5 μM, 10 μM), and ETC Complex I activity was determined. C4-2B STS cells treated with 10 μM of SI-2 had the most significant reduction in ETC complex 1 activity compared to lower doses and control (Figure 3C,D).

**Figure 4:**
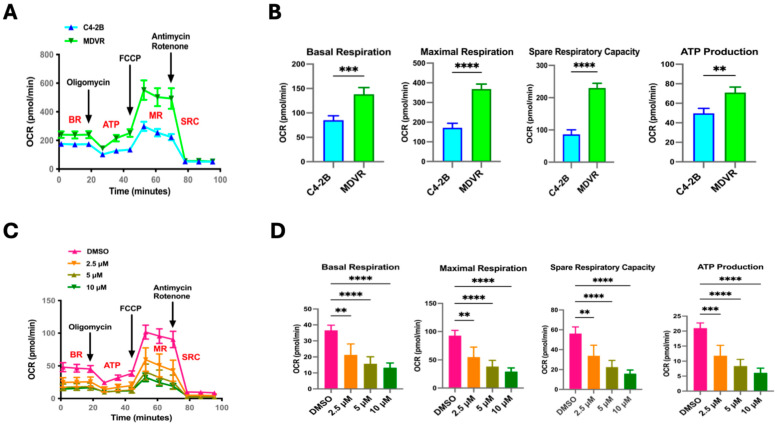
STS inhibitor (SI-2)-treated enzalutamide-resistant cells (MDVR) display decreased mitochondrial functionality. (**A**) Seahorse Extracellular Flux analyzer, Mito Stress Test assay results comparing parental C4-2B and enzalutamide-resistant C4-2B cells (MDVR). Mito Stress Test summary represents differential OCR between naive and resistant cells after the injection of respiration-modulating compounds oligomycin, carbonyl cyanide-4-(trifluoromethoxy) phenylhydrazone (FCCP), rotenone, and Antimycin. (**B**) The quantification of relative OCR levels in C4-2B and MDVR cells shown as basal respiration (BR), maximal respiration (MR), spare respiratory capacity (SRC), and ATP production (ATP). (**C**) Mito Stress Test output demonstrating differential mitochondrial respiration in MDVR cells treated with SI-2 (2.5 μM, 5 μM, and 10 μM, respectively). (**D**) The relative values of BR, MR, SRC, and ATP derived from the overall OCR of MDVR cells treated with SI-2 (2.5 μM, 5 μM, and 10 μM, respectively). ** *p* < 0.01, *** *p* < 0.005, and **** *p* < 0.001.

Having previously demonstrated that STS overexpression confers resistance to Enza; next, we attempted to compare mitochondrial function between parental C4-2B cells and our established Enza-resistant CRPC cells (MDVR) [10]. Given that our group reported upregulation in the mitochondrial-related gene signaling pathways in our MDVR cells, we subjected the C4-2B and MDVR cells to the Mito Stress Test. We observed MDVR cells with significantly higher overall OCR rates in comparison to parental C4-2B cells (Figure 4A). This was also reflected by elevated BR, MR, SRC, and ATP production levels in MDVR cells compared to C4-2B (Figure 4B). We next determined if targeting STS in MDVR cells could affect mitochondrial respiration. We used the Mito Stress Test on MDVR cells treated with SI-2 doses (2.5 μM, 5 μM, 10 μM). Overall, we observed significantly hampered mitochondrial activity in all SI-2 doses (Figure 4C). Specifically, BR, MR, SRC, and ATP production were significantly reduced in the STS-targeted groups compared to the DMSO-treated control cells (Figure 4D).

**Figure 5:**
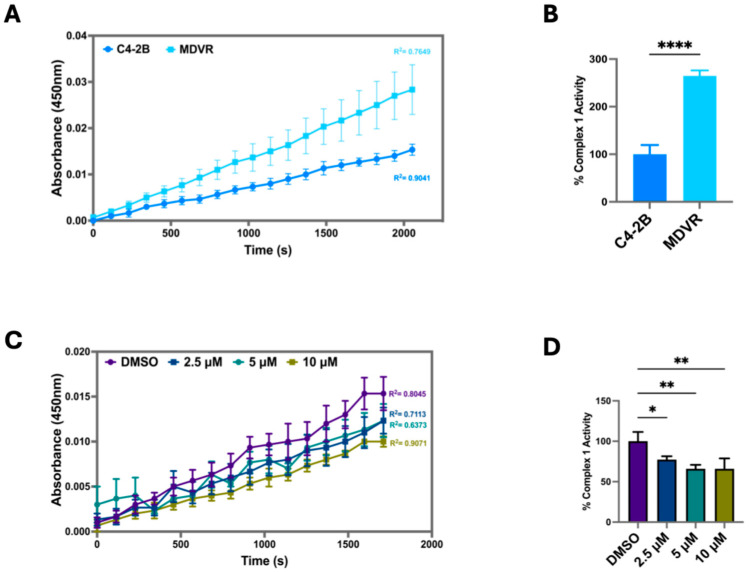
Inhibition of STS in enzalutamide-resistant cells (MDVR) attenuates mitochondrial OXPHOS Complex I enzyme activity. (**A**) Electron Transport Chain (ETC) Complex 1 enzyme activity was determined in parental C4-2B and enzalutamide-resistant C4-2B cells (MDVR). Cell culture extracts were processed and added to a microplate to measure optical density at 450 nm over time. (**B**) The derived % Complex 1 Activity was calculated in C4-2B and MDVR cells by using the following formula: % Complex 1 Activity = (MDVR group linear rate of increase in absorbance at OD 450 nm over time/C4-2B group linear rate of increase in absorbance at OD 450 nm over time) × 100%. (**C**) ETC Complex 1 activity was determined in MDVR cells treated with SI-2 (2.5 μM, 5 μM, 10 μM, respectively) at OD 450 nm over time. (**D**) The slope of the linear rate of increase in absorbance at OD 450 nm over time was used to derive % Complex 1 activity in MDVR cell treated with SI-2 (2.5 μM, 5 μM, and 10 μM, respectively). * *p* < 0.05, ** *p* < 0.01, and **** *p* < 0.001.

Given the significant differences in readings from the Mito Stress Test between C4-2B and MDVR cells, we wanted to test the ETC complex 1 activity in these lines. Upon completion of the activity assay, we found MDVR cells to have significantly elevated ETC complex 1 activity in comparison to C4-2B (Figure 5A,B). Additionally, SI-2 treatment in MDVR cells at all doses (2.5 μM, 5 μM, 10 μM) significantly depleted complex 1 activity (Figure 5C,D). Taken together, these results reveal the robust mitochondrial function of C4-2B STS and MDVR cells, indicating a potential role of STS in allowing the mitochondria-based adaptive capacity of cancer cells and the promotion of resistance to enzalutamide treatment.

### 3.3. Elevated STS Induces DHEAS-Mediated Organoids Formation

**Figure 6:**
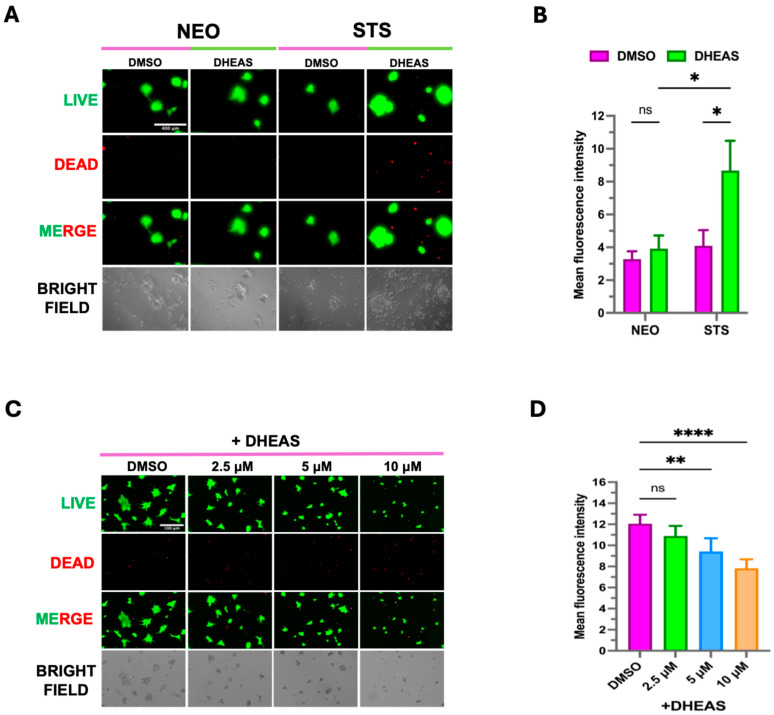
DHEAS supplementation enhances organoid growth in C4-2B STS cells. (**A**) C4-2B NEO (NEO) and C4-2B STS (STS) PDX tumor tissues were harvested and dissociated to form 3-D organoids in Matrigel. Organoids were treated with DMSO and 100 nM of DHEAS for 14 days. A LIVE/DEAD^®^ Viability/Cytotoxicity Assay Kit was used to visualize the viability of organoids. The scale bar is 400 μm. NEO is the control. (**B**) Organoid fluorescence intensity from LIVE/DEAD staining was quantified by FIJI software (Version 1.54). (**C**) C4-2B NEO (NEO) and C4-2B STS (STS)-cultured cells were used to form 3D organoids in Matrigel. Organoids were treated with DMSO, 100 nM of DHEAS, and SI-2 (2.5 μM, 5 μM, and 10 μM, respectively) for 14 days. The LIVE/DEAD^®^ Viability/Cytotoxicity Assay Kit was used to visualize the viability of organoids. The scale bar is 100 μm. (**D**) Organoid fluorescence intensity from LIVE/DEAD staining was quantified by FIJI software. * *p* < 0.05, ** *p* < 0.01, and **** *p* < 0.001.

Given that STS converts DHEAS to DHEA, we wanted to determine if STS overexpression and DHEAS media supplementation would affect organoid formation. C4-2B NEO and C4-2B STS tumor material from mice was used to derive a single-cell suspension. In a mixture with Matrigel, the single-cell suspension formed NEO and STS organoids in a 96-well plate. We observed the C4-C4-2B STS organoids supplemented with DHEAS to grow significantly larger compared to the controls (Figure 6A,B). Moreover, we wanted to assess the effects of the SI-2 (2.5 μM, 5 μM, 10 μM) treatment on C4-2B STS organoids. Using C4-2B STS 2D-cultured cells and Matrigel, we formed C4-2B STS organoids in a 96-well plate. We subjected the STS organoids to SI-2 doses (2.5 μM, 5 μM, 10 μM) and DHEAS (100 nM). SI-2 at doses of 5 μM and 10 μM significantly hindered C4-2B STS organoid growth despite the DHEAS addition (Figure 6C,D). These data demonstrate that DHEAS-driven cell growth and organoid viability are promoted by STS overexpression and that SI-2 is effective at inhibiting this enhanced STS activity.

## 4. Discussion

Almost one hundred years ago, it was discovered by Otto Warburg that cancer cells alter their metabolism to preferentially use the glycolytic pathway, despite oxygen availability [30,31]. This metabolic shift was the focus of cancer metabolism for decades. Currently, it is understood that many mitochondrial processes, such as modified oxidative phosphorylation, are essential in tumor growth and metastasis [32,33]. Given the importance of metabolic reprogramming in cancer advancement, the efforts are now on precise treatments to target metabolic pathways in cancer therapy and drug resistance [34,35,36,37,38,39].

Our study discovered that STS overexpression and enzalutamide treatment enhance mitochondrial respiration and Electron Transport Chain Complex I activity, whereas suppressing STS activity with SI-2 significantly reduces oxidative phosphorylation in treatment-resistant CRPC cells. These findings are consistent with previous studies that demonstrate upregulated oxidative metabolism in enzalutamide-resistant PCa, but to our knowledge, we are the first to reveal a relationship between increased STS expression and heightened mitochondrial functionality [40,41]. We demonstrate that STS could be an effective target in improving enzalutamide treatment in CRPC patients.

In our prior research, we showed that STS inhibition results in the reduced expression of AR signaling genes in CRPC cells [10]. It is well known that AR activation modulates many metabolic pathways, such as glucose metabolism, the citric acid cycle, and oxidative phosphorylation, to support cell proliferation in PCa [32,42,43,44,45]. Given that STS is critical to androgen production and plays a role in the AR signaling pathway, it is likely that STS overexpression results in enhanced AR transcriptional activity, mediating metabolic reprogramming in cancer cells [9,10]. Additionally, we previously demonstrated that upregulated STS activity contributes to amplified DHEA production [10]. The potential of DHEA as a mitochondrial energy modulator is highlighted by many studies, which have emphasized its role in glucose uptake and metabolism, mitochondrial structural alterations, reactive oxygen species (ROS) generation, and oxidative phosphorylation [12,16,46,47,48,49,50]. For example, in human neuroblastoma cells, DHEA treatment increased cellular viability in glucose deprivation conditions by exerting protective effects [51]. Similarly, in human oocyte granuloma HO23 cells, DHEA treatment decreased mitochondrial ROS and restored mitochondrial membrane depolarization upon starvation-induced apoptosis conditions [52]. In the rat brain, DHEA increased mitochondrial ATPase activity [12,53]. In our current study, we present evidence for Myc signaling enrichment in STS overexpression cells. It has been extensively studied that Myc can directly control genes involved in glycolysis and oxidative phosphorylation [54,55,56,57,58,59]. Given this known interaction, increased Myc signaling may contribute to the enhanced oxidative phosphorylation phenotype observed in our study. Overall, our findings highlight an STS-driven enhanced mitochondrial metabolism in CRPC.

Based on our study, metabolic inhibitors, in conjunction with NGATs, could enhance treatment outcomes by disrupting pathways supporting tumor growth. In support of this approach, it would be impactful to explore how STS overexpression and inhibition affect oxidative phosphorylation when cells face high energy demands. Further research is needed to investigate the mechanisms by which STS interacts with these metabolic pathways.

In summary, we found that STS overexpression enhances mitochondrial signaling, mitochondrial respiration, and ETC Complex I activity. Inhibiting STS activity by using SI-2 suppresses mitochondrial OCR, ETC Complex I activity, cellular viability, and organoids formation. These studies suggest that STS has a key function in regulating metabolic activity in CRPC, and future research on inhibiting this enzyme in combination with existing metabolic treatments could help overcome therapeutic resistance.

## 5. Conclusions

This study suggests that STS expression alters mitochondrial oxidative phosphorylation in CRPC by increasing Electron Transport Chain Complex I activity. Our observations highlight STS as a key player in enhanced energy metabolism and therefore drug resistance in advanced PCa progression. Our data further supports STS as a target for cancer therapy.

## Data Availability

The raw data generated for this study will be made available by the corresponding author upon request.

## Abbreviations

The following abbreviations are used in this manuscript:

PCa	Prostate Cancer
AR	Androgen Receptor
NGATs	Next-Generation Anti-Androgen Therapies
CRPC	Castration-Resistant Prostate Cancer
STS	Steroid Sulfatase
RNA-Seq	RNA-Sequencing
Enza	Enzalutamide
Abi	Abiraterone
Daro	Darolutamide
Apa	Apalutamide
OCR	Oxygen Consumption Rate
BR	Basal Respiration
SRC	Spare Respiratory Capacity
MR	Maximal Respiration
ATP	ATP Production
DHEA	dehydroepiandrosterone
DHEAS	Dehydroepiandrosterone sulfate
GSEA	Gene Set Enrichment Analysis
ETC	Electron Transport Chain
ROS	Reactive Oxygen Species
